# *In-vitro* flow assessment study of intra-saccular endovascular devices for brain aneurysm treatment: SEAL™ vs. WEB™

**DOI:** 10.3389/fneur.2026.1830746

**Published:** 2026-06-19

**Authors:** Hamid Mansouri, Nedim Gulkaya, Matthew J. Gounis, Mohammad AlMajali, Amit Chaudhari, Darwin G. Ramirez Abreu, Syed F. Zaidi, Yazan K. Ashouri, Naoki Kaneko, Brian T. Jankowitz, David J. Altschul, Boris Pabon, Pervinder Bhogal, Thomas J. Wolfe, Edgard L. Pereira, Aamir Badruddin, Laila Ibrahim, Omid Amili, Osama O. Zaidat

**Affiliations:** 1 Department of Mechanical, Industrial, and Manufacturing Engineering, University of Toledo, Toledo, OH, United States; 2 Galaxy Therapeutics Inc., Milpitas, CA, United States; 3 Department of Radiology, University of Massachusetts Chan Medical School, Worcester, MA, United States; 4 Mercy Health Neuroscience Institute, Neurology and Neurosurgery, Toledo, OH, United States; 5 Department of Neurology, University of Toledo, Toledo, OH, United States; 6 Westwood Imaging Center & Interventional Radiology Clinic, Los Angeles, CA, United States; 7 Hackensack Meridian Neuroscience Institute at JFK University Medical Center, Edison, NJ, United States; 8 Montefiore Einstein Advanced Care Neurosurgery, Elmsford, NY, United States; 9 Centro Comercial Cauca Centro, Universidad de Antioquia, Antioquia, Colombia; 10 Department of Interventional Neuroradiology, The Royal London Hospital, Barts NHS Trust, London, United Kingdom; 11 Aurora Neuroscience Innovation, Milwaukee, WI, United States; 12 Neuro Endovascular Surgery, McLeod Medical Center, Florence, SC, United States; 13 Powers Health Medical Group, Neurosurgery and Spinal Surgery, Munster, IN, United States

**Keywords:** aneurysm, *in vitro* PIV, intrasaccular devices, patient-specific, SEAL and WEB

## Abstract

**Background:**

Intrasaccular devices such as SEAL™ and WEB™-SL are designed to disrupt aneurysmal flow. We present a new *in vitro* method for estimating neurointerventional devices’ flow dynamics.

**Methods:**

Particle image velocimetry (PIV) method was used in a patient-specific middle cerebral artery (MCA) wide-neck bifurcation aneurysm (WNBA) model to measure velocity, residence time (RT), and vorticity strength (VS). Tested devices included 3 each of SEAL-BASE (6 × 2 mm), SEAL-ARC (6 × 5 mm), and WEB-SL (6 × 4 mm).

**Results:**

Twelve experiments were performed (3 unique devices per size and 3 controls); the mean RT was 0.042 ± 0.006 s (control) and increased with SEAL-BASE (0.387 ± 0.128), SEAL-ARC (0.33 ± 0.092), and WEB-SL (0.171 ± 0.034) (all *p* < 0.05 vs. control), and was longer in SEAL-BASE (*p* = 0.046) and SEAL-ARC (*p* = 0.041) than WEB-SL, with no difference between SEAL configurations (*p* = 0.30). The VS decreased from 66.48 ± 8.58 1/s (control) to 14.06 ± 2.80, 13.94 ± 2.53, and 27.60 ± 4.16 for SEAL-BASE, SEAL-ARC, and WEB-SL, respectively. The SEAL devices differed from WEB-SL (*p* = 0.007, 0.006) but not from each other (*p* = 0.52). Velocity magnitude dropped from 109.4 ± 15.5 mm/s (control) to 12.42 ± 3.8, 14.2 ± 3.5, and 25.34 ± 3.16 for SEAL-BASE, SEAL-ARC, and WEB-SL, respectively; greater reductions with SEAL-BASE (*p* = 0.006) and SEAL-ARC (*p* = 0.007) versus WEB-SL, with no difference between the two SEAL configurations (*p* = 0.29).

**Conclusion:**

Both SEAL configurations achieved greater flow reduction compared to the WEB-SL of similar width in the same aneurysm model. Results highlight the unique utilization of the PIV method for the mechanistic decoupling of momentum transfer into the aneurysm sac resulting from intra-aneurysmal mesh structures and point toward their potential for further neurointerventional device optimization.

## 1 Introduction

Flow diverter stents provide a minimally invasive alternative to traditional treatment methods for intracranial aneurysms (IA), such as primary coiling, adjunctive device-assisted coiling, or surgical clipping (1). However, treating wide-neck bifurcation aneurysms (WNBA) remains a challenge for endovascular intraluminal flow diverting techniques due to complex stent configurations and the risk of covering bifurcation branches. These limitations have driven the development of intrasaccular flow disruptors for WNBA (2). Among the available devices, the Woven EndoBridge (WEB™) is the only FDA-approved device (3). However, its current limitation includes a modest rate of 1-year occlusion of 53.8% (4). To address these limitations, the Saccular Endovascular Aneurysm Lattice Embolization System (SEAL™), developed by Galaxy Therapeutics, Inc. (Milpitas, CA, USA), represents a next-generation device. The SEAL system features a self-expanding, dual-layer braided implant with a proprietary process to increase flow diversion, aiming at improving aneurysm occlusion rate (5, 6).

Alterations in flow dynamics by these intrasaccular devices are fundamental contributors to diverting and disrupting the flow inside the aneurysm sac. Multiple hemodynamic metrics have been proposed to assess device performance, but *in vivo* measurement in patients remains challenging and costly (7).

The objective of this study is to use the particle image velocimetry (PIV) method for flow characterization with intra-saccular devices. *In vitro* flow experiments using PIV enable the study of controlled flow conditions with high spatial and temporal resolution, which relies on the cross-correlation of consecutive image pairs to obtain Eulerian velocity fields (8). In this study, the PIV flow metrics were compared between the SEAL and the WEB device.

## 2 Methodology

### 2.1 Model specifications

**Figure 1:**
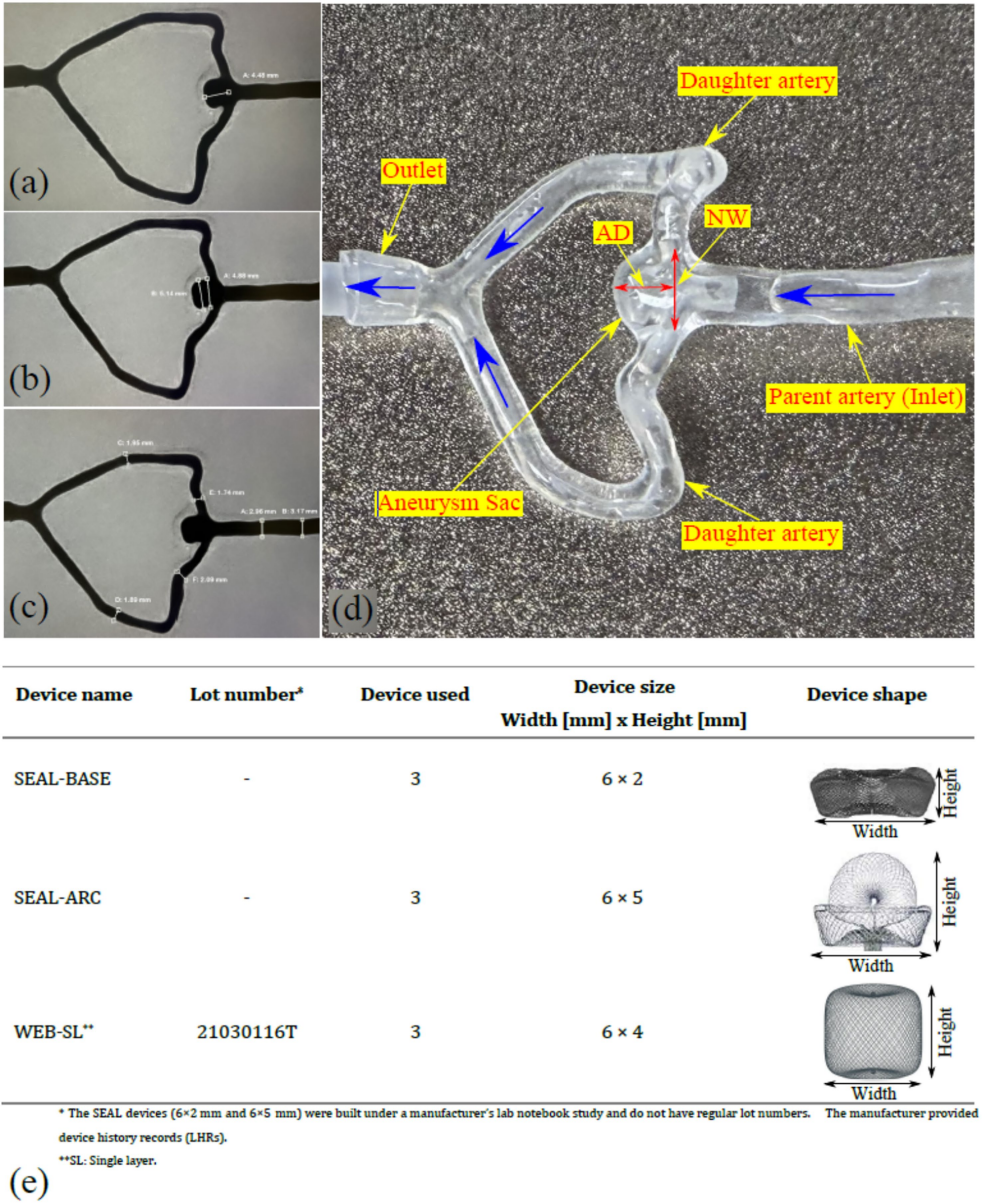
Aneurysm silicon model and medical devices used. DSA image providing the aneurysm’s morphology with vessel dimensions provided by Galaxy Therapeutics: **(a)** Aneurysm depth (AD), **(b)** Aneurysm width and neck width (NW), **(c)** The parent and daughter vessels’ inner diameters, **(d)** Reconstructed silicon model with an MCA aneurysm sac (patient-specific) provided by Galaxy Therapeutics. **(e)** A summary of the medical device identification and size used in this study.

A patient-specific model of a middle cerebral artery (MCA) aneurysm was used, with digital subtraction angiography (DSA) to confirm measurement as in Figures 1a–d.

The silicone model WNB MCA aneurysm measured 5.14 mm × 4.48 mm in width and depth, while 4.88 mm in neck. The dome-to-neck ratio was 1.1 and an aspect ratio of 0.92 (Figure 1d). The inflow (parent artery) and outflow directions, aneurysm depth (AD), and neck width (NW) used to analyze flow conditions in the sac and adjacent daughter arteries are also indicated in this figure.

### 2.2 Devices used

Figure 1e provides an overview of intrasaccular endovascular devices utilized in the experiments. A total of 12 experiments were performed: three repeated control measurements (without any devices), three SEAL-BASE, three SEAL-ARC, and three WEB-SL. These repetitions account for potential variations in results due to human handling during deployment in the aneurysm sac.

### 2.3 Controlled flow loop

The aneurysm model was integrated into a custom circuit capable of reproducing physiologic steady and pulsatile flows. Further details regarding the experimental setup are provided in Supplementary Figure S1 (35). A frequency-controlled centrifugal pump regulated flow, measured with a Transonic ME9PXL (0–10 LPM, ±10%) flow meter and precision pressure transducer. A CW5000 800 W chiller maintained a constant temperature, and valves directed the flow. The test section housed the aneurysm model on dual supports, with a medical Y-connector as the deployment site. Endovascular neurosurgeons deployed and retrieved SEAL and WEB devices using compatible microcatheters and electrical detachment systems to ensure clinical accuracy. Flow entered the model inflow, divided between daughter branches, and exited through the outflow (Figure 1d).

A steady inflow rate of Q₀ = 85 mLPM (≈ 320 mLPM scaled to blood) was applied to the aneurysm model, corresponding to parent-vessel velocities of 200 mm/s (750 mm/s under blood flow conditions). These conditions matched a previous evaluation of 10 endovascular devices from Galaxy Therapeutics (9) and were based on 4D Flow MRI data of the circle of Willis (10).

Experiments were conducted at ~20 °C with water (*ν* ≈ 1 × 10^−6^ m^2^/s, *ρ* ≈ 998 kg/m^3^). Accounting for blood’s higher viscosity (3.77 cSt vs. 1 cSt), flow was scaled to a Reynolds number of approximately 600, which is consistent with values typically observed in cerebral vessels. The use of a mixture of water and glycerol to match blood viscosity is also possible, as opposed to using water alone. However, it would yield the same results with more difficulties in fluid handling (11, 12). Based on the previous relevant studies, the Newtonian assumption is appropriate in intracranial arteries, provided the Reynolds number remains within the laminar flow range (Re < 2,000), as is typical in most intracranial arteries (13).

### 2.4 Optical imaging

The flow was seeded with 27–32 *μ*m neutrally buoyant fluorescent tracer particles. Particle inertia, quantified by the Stokes number (*Stk* = *τ_p_/τ_f_*, where *τ_p_* = *ρ_p_d*^2^*_p_/*(18 *μ*) and *τ_f_* = *D/U*), was ~0.0033, confirming that tracers accurately followed the flow (14). Tracers were neutrally suspended in water and injected using a precision syringe pump. Four pulsed 415 nm LED lights illuminated the field, while a high-speed camera (PHANTOM T1340, 2,048 × 1,952 pixels, 13.5 μm sensor pixel size) with a ZEISS Milvus 135 mm f/2 lens captured the particle motion at 1,200 fps, with a maximum capability of 3,180 fps at full resolution. A MIDOPT Bi518-77 optical filter and Nikon PK-13 extension ring enhanced the signal-to-noise ratio.

For each case, 3,000 frames were recorded and processed in PIVView 3.9 software to calculate the local velocity with high spatial and temporal resolution. The software utilized the Least Squares Gaussian fit, specifically over a 3 × 3 point grid, to identify the peak intensity of each particle image accurately. This method enhances the precision of particle center localization by fitting a Gaussian model to the intensity distribution around each detected peak. A Fast Fourier Transform (FFT)-based method was employed for cross-correlation analysis, utilizing a 32 × 32 pixel interrogation window with an initial sampling window of 64 × 64 pixels. This size was chosen to balance spatial resolution and statistical reliability in velocity calculations. The substantial 75% overlap between adjacent interrogation windows was critical for increasing the spatial resolution of the velocity field measurements and ensuring continuity in the observed flow patterns (15).

## 3 Results

### 3.1 Flow fields

**Figure 2:**
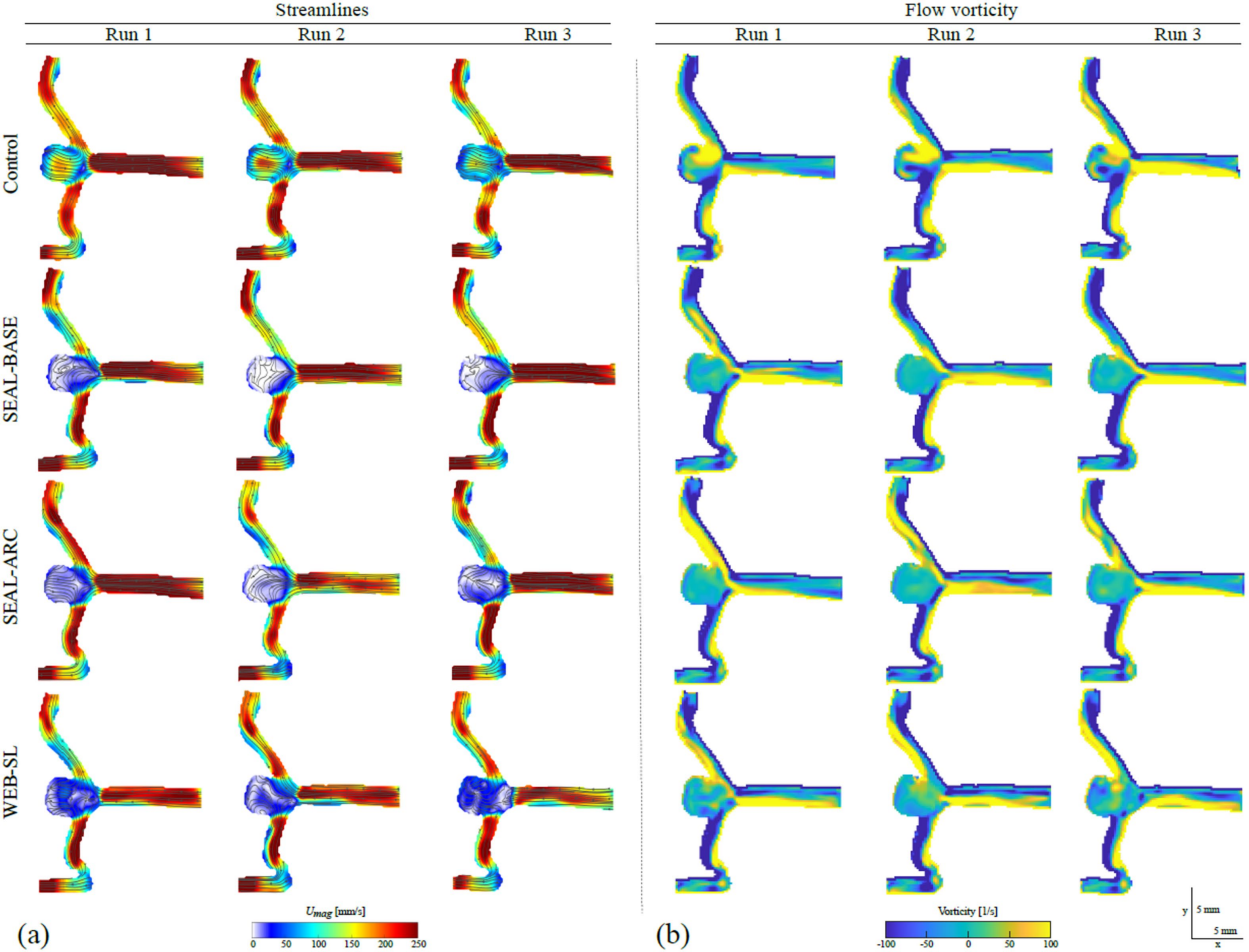
Two-dimensional flow maps within the aneurysm model. **(a)** Velocity magnitude overlaid with streamlines for the control and device-deployed cases, averaged over 3,000 frames. **(b)** Corresponding vorticity distributions illustrating rotational flow intensity within the sac. Each column represents one of three experimental repetitions per case.

Figure 2a shows the flow pattern within the aneurysm sac and parent vessels, with velocity magnitude ( $U_{\mathrm{mag}}=\sqrt{u_{x}^{2}+u_{y}^{2}}$ ) contours overlaid by streamlines to visualize flow structures, where *u_x_* and *u_y_* denote the *x*- and *y*-direction velocity components. Velocity vectors (Supplementary Figure S2) are spaced at ~0.2 mm, displaying every other vector for clarity, and averaged over 3,000 frames. The parent vessel exhibits high-speed flow with a dominant vortex inside the sac. The top row represents the control (no device), followed by SEAL-BASE, SEAL-ARC, and WEB-SL deployments, each tested in triplicate.

Figure 2 and Supplementary Figure S2 show highly consistent results across three repetitions, confirming measurement reliability. Device deployment markedly disrupted the intra-sac flow, with the degree of disturbance varying by device. The SEAL devices (second and third rows) demonstrated greater suppression of intra-sac flow compared to the WEB-SL (last row). Figure 2b presents vorticity contours showing similar repeatability. The control case exhibited high sac vorticity, while all devices reduced vortical flow, with SEAL devices achieving stronger vorticity attenuation than WEB-SL.

### 3.2 Flow parameters

#### 3.2.1 Mean residence time

Residence time plays a crucial role in aneurysm treatment effectiveness (16). Residence time reflects regions of prolonged intra-aneurysmal flow recirculation. Increased RT following device placement promotes thrombus formation and aneurysm occlusion by enhancing flow stagnation near the aneurysm wall, which is a key mechanism underlying aneurysm occlusion and stabilization (17, 18).

**Figure 3:**
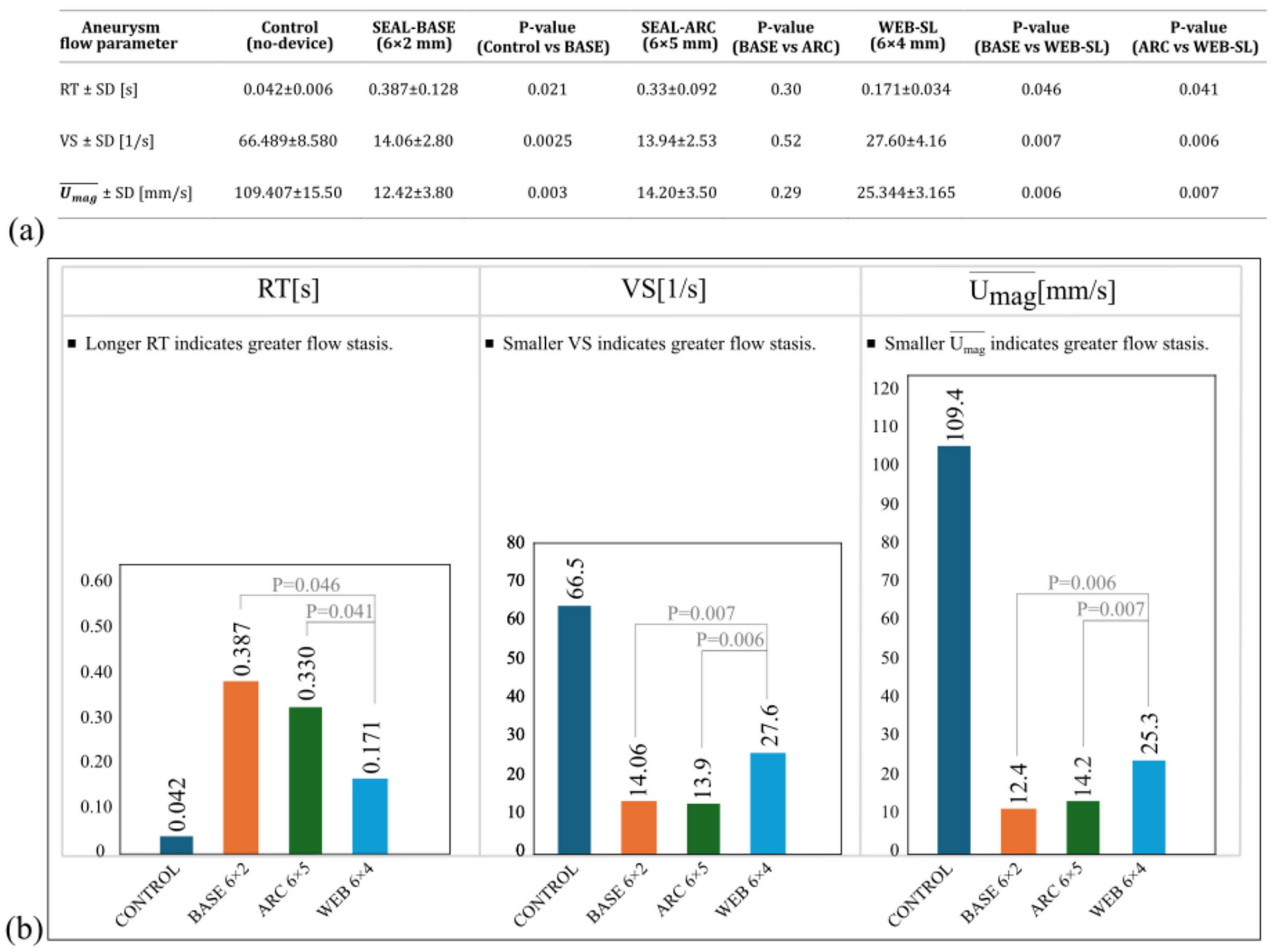
Comparison of intra-aneurysmal hemodynamic parameters across control (no-device) and device-deployed conditions. **(a)** Table summarizing mean values ±SD and statistical comparisons for mean residence time (RT), mean vorticity strength (VS), and mean velocity magnitude ( $\bar{U_{\mathrm{mag}}}$ ). **(b)** Bar plots representing the corresponding tabulated data with annotated values and statistical comparisons between devices.

In this study, the mean residence time metric (RT) was estimated using the 2D form of the equation from the study by Li et al. (19) based on the Eulerian approach. We denote this metric as *RT*_sac_, which is defined as $\;RT_{\mathrm{sac}}=\frac{D_{\mathrm{sac}}}{U_{\mathrm{sac}}},$ where *D*_sac_ represents the estimated sac depth, as shown in Figure 1a, measured as 4.48 mm from angiography imaging, and *U*_sac_ is the mean velocity magnitude within the region of interest of the sac ( $U_{\mathrm{sac}}=\bar{U_{\mathrm{mag}}}$ as reported in the table of Figure 3a). For mean velocity ( $\bar{U_{\mathrm{mag}}}$ ), SEAL-BASE produced a significant reduction compared with the control (*p* = 0.003). The difference between SEAL-BASE and SEAL-ARC was not significant (*p* = 0.29). However, when comparing the SEAL-BASE with the WEB-SL device, a significant decrease in velocity was observed with SEAL-BASE (*p* = 0.006).

Figure 3a presents the table in which the first row shows the RT values expressed as mean ± SD in seconds. Compared to the control case, all devices significantly increased residence time, indicating reduced flow velocity and enhanced flow stagnation. The SEAL-BASE device exhibits the highest RT, followed by SEAL-ARC with a slightly lower RT value, while WEB-SL demonstrates the lowest RT among the three. This observation suggests that SEAL devices may be more effective in prolonging flow residence time within the aneurysm sac, which could enhance thrombus formation and facilitate aneurysm occlusion. It is suggested in the literature that once the clotting process is triggered, the thrombus is likely to form in the regions of slow-moving flow, low shear stress, and thus in the increased residence time regions, e.g., see Liu et al. (20).

In this study, the longest mean value of RT was found for the SEAL-BASE, which also showed a low level of vortical motion, suggesting it was the most effective device in treatment (based on these two metrics), as it reduces the flow inside the aneurysm sac better when compared to other prototypes and the control case.

The comparison of mean RT values across devices shows that the SEAL-BASE exhibited a significantly longer RT compared with the control (*p* = 0.021). In contrast, the difference between SEAL-BASE and SEAL-ARC was not statistically significant (*p* = 0.30). However, when comparing SEAL-BASE with the WEB-SL, a significant reduction in RT was observed (*p* = 0.046).

It is worth noting that several definitions of the flow residence time exist in the literature, as reported by Shadden and Arzani (21). Based on the definition of RT, the values range differently. We used an Eulerian approach, as shown by Li et al. (19), which is an effective metric and strongly correlates with Lagrangian methods in aneurysmal flow settings.

#### 3.2.2 Flow vorticity

Vorticity, a fundamental metric in fluid dynamics, quantifies the local rotational motion of fluid elements within the flow field (22). In aneurysm hemodynamics, persistent vortices can increase residence time and elevate rupture risk (23). The deployment of intra-saccular devices aims to mitigate vorticity strength and associated hemodynamic forces, thereby promoting thrombus formation (24). In this study, vorticity was used to characterize rotational flow patterns within the aneurysm sac and to evaluate the effectiveness of each device in altering intra-sac circulation.

By analyzing the vorticity field within the aneurysm sac, we evaluated how effectively intrasaccular devices disrupted high-vorticity regions, which indicate fluid rotation. Reduced vorticity corresponds to flow stagnation, which promotes thrombus formation and inhibits aneurysm growth. The second row of Figure 3a presents mean vorticity strength (VS ± SD) for all devices and the control. As expected, the control aneurysm showed the lowest mean of RT and the highest vorticity. Among devices, the two SEAL models, which had the highest RT values, also showed lower (favorable) vorticity levels compared to the WEB-SL. device.

All statistical analyses were conducted using JMP software (version 13; SAS Institute Inc., Cary, NC, USA). Continuous variables were summarized as mean ± SD. Device comparisons were performed using the two-tailed Student *t*-test, and statistical significance was evaluated at the 95% confidence level.

For vorticity, a significant reduction was observed when comparing SEAL-BASE with the control (*p* = 0.0025). The difference between SEAL-BASE and SEAL-ARC was not significant (*p* = 0.52). However, a significant reduction in vorticity was noted when comparing SEAL-BASE to the WEB-SL device (*p* = 0.007).

A study by Lieber et al. (25) supports that placement of low-porosity stents across the aneurysm orifice reduces vortex speed and flow interaction, with significant flow vorticity reduction that promotes thrombus formation. Moreover, some other studies, such as Biasetti et al. (26) and Tupin et al. (27), revealed that reduced vorticity correlates with more favorable thrombus formation. Vortical structures influence platelet dynamics, suggesting a potential link between flow vorticity and thrombus development in aneurysms.

Figure 3b is a representative figure of the data listed in Figure 3a, illustrating the hemodynamic comparison of control flow conditions with three intrasaccular devices. The results show a substantial reduction in vorticity and velocity magnitude for all devices relative to control, indicating disruption of intra-aneurysmal flow. The mean residence time exhibited greater variability, with SEAL-BASE producing the highest increase, followed by SEAL-ARC, while WEB-SL resulted in the lowest value among the devices. These findings highlight differences in the flow-modifying effects of each device design while considering two flow metrics (RT and VS).

## 4 Discussion and conclusion

Hemodynamics is widely recognized as a key factor in the initiation, progression, and potential stabilization or rupture of cerebral aneurysms (28). A deeper understanding of these mechanisms is critical for advancing both the diagnosis and treatment of intracranial aneurysms. However, utilization of PIV as an *in vitro* testing for early device development and prototype evaluation, or comparing the same devices in the same class, is not well established. Only a limited number of PIV studies have investigated the hemodynamic impact of intracranial flow diverters, providing their influence on intra-aneurysmal flow dynamics (29, 30), but not intrasaccular flow-disrupting devices, which remain less explored experimentally.

This study used *in vitro* PIV in a patient-specific MCA WNBA model to evaluate flow dynamics before and after implantation of comparable WEB-SL, SEAL-BASE, and SEAL-ARC devices. The experiments showed high repeatability, and all devices significantly reduced mean velocity magnitude and vorticity while increasing residence time, consistent with prior studies on flow diversion strategies (31). In all flow metrics, the SEAL devices achieved greater flow reduction compared to the WEB-SL of similar width in the same aneurysm model. The comparative flow analysis demonstrated that SEAL BASE and ARC reduced vorticity strength by 78.9 and 79.0%, respectively, while WEB achieved a 58.4% reduction relative to control. Similarly, mean sac velocity was substantially decreased by 88.7% for SEAL BASE, 87.0% for SEAL ARC, and 76.8% for WEB, indicating greater flow stagnation with the SEAL devices. Correspondingly, RT increased 9.2-fold for BASE, 7.9-fold for ARC, and 4.1-fold for WEB versus control, suggesting more favorable intra-sac flow retention conducive to thrombus formation. Overall, these findings highlight the enhanced hemodynamic performance of the SEAL devices in promoting flow disruption and stabilization compared to the WEB implant. Clinical findings from a recent SEAL intrasaccular flow disruption study further support the translational relevance of these hemodynamic observations. In a cohort of 13 wide-neck bifurcation aneurysms, SEAL demonstrated 100% technical success with no major periprocedural strokes, subarachnoid hemorrhages, or neurologic deaths. At 12-month follow-up, complete occlusion was achieved in 84.6% of cases and adequate occlusion in 92.3%, indicating promising safety and efficacy outcomes for the treatment of WNBAs (32).

This study establishes a framework for quantifying hemodynamic parameters within an *in vitro* cerebral aneurysm model and provides a foundation for future validation against animal and clinical data to improve translational relevance to aneurysm thrombosis and occlusion outcomes.

A precise understanding of patient-specific flow dynamics following endovascular treatment is of critical clinical significance. It enables clinicians to assess the suitability of aneurysms for intra-saccular endovascular flow diverter device therapy and predict post-treatment outcomes. Moreover, these insights contribute to a deeper understanding of the pathophysiological mechanisms underlying aneurysms that are either refractory to treatment or prone to rupture despite flow diversion (33, 34).

The hemodynamic alterations induced by these devices are only partially understood here, necessitating further investigations into their influence on intra-aneurysmal flow patterns in animal and human studies. A more comprehensive understanding of these effects is essential for optimizing treatment strategies, improving patient selection criteria, and enhancing surgical planning for endovascular aneurysm repair.

Several limitations should be considered when interpreting these findings. First, this study evaluated a single patient-specific MCA wide-neck bifurcation aneurysm model, which may limit generalizability to aneurysms with different morphologies and hemodynamic conditions. In addition, although Reynolds-number-scaled water was used to approximate physiologic flow, it does not fully reproduce the rheological and biological properties of blood. Therefore, the results should be viewed as a controlled comparative assessment of device-induced flow disruption rather than a direct predictor of clinical outcomes. Future studies should incorporate multiple aneurysm models, blood-analog fluids, and clinical correlation analyses.

## Data Availability

The original contributions presented in the study are included in the article/Supplementary material; further inquiries can be directed to the corresponding authors.
