# Supplementary material for: In-vitro flow assessment study of intra-saccular endovascular devices for brain aneurysm treatment: SEAL™ vs. WEB™
Source: Front Neurol. 2026 Jun 19;17:1830746. doi: 10.3389/fneur.2026.1830746 (PMC13327948; doi:10.3389/fneur.2026.1830746)
Supplement: Supplementary file 1 [file Data_Sheet_1.PDF]

Supplementary Material

Supplementary Figures

The flow loop was established in the Mechanical, Industrial, and Manufacturing Engineering (MIME) Department at the University of Toledo and equipped with optical diagnostic instrumentation for real-time particle tracking in high-resolution vascular models (Mansouri, 2024).

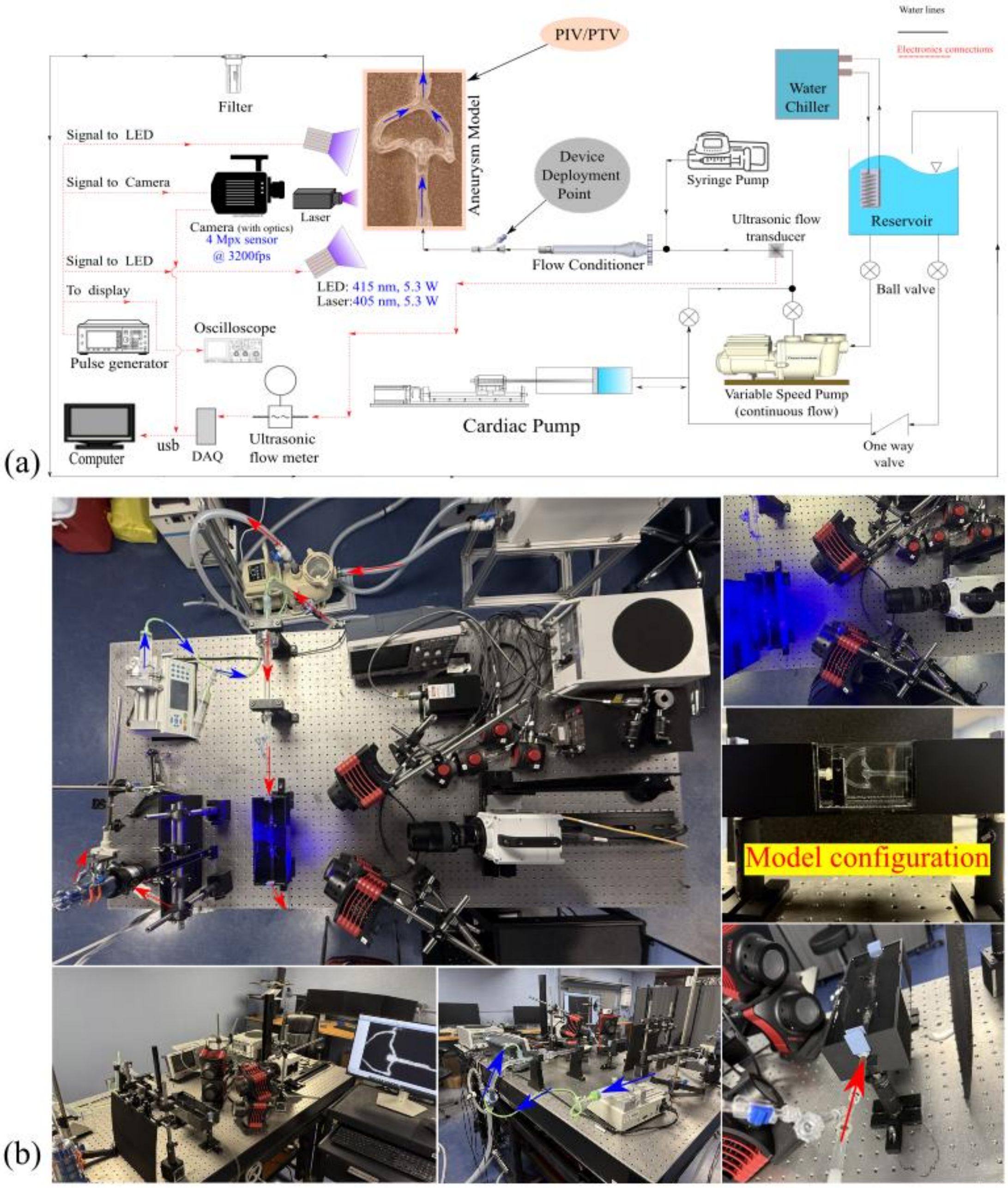

Figure S1: Experimental setup for optical imaging. (a) Schematic of the universal configuration developed for PIV/PTV experiments. (b) Principal components of the setup used for optical imaging measurements.

Figure S1 a shows the flow-loop schematic for in-vitro PIV/PTV, and Figure S1 b illustrates optical-velocimetry components. Water was used as the working fluid and dynamically scaled to blood-equivalent conditions by matching the Reynolds number (Re = UD/ν) between in-vitro and physiological flows.

Figure S2 presents flow field representations with velocity vectors superimposed on velocity contours, color-coded by local velocity magnitude, for control and device-deployed conditions.

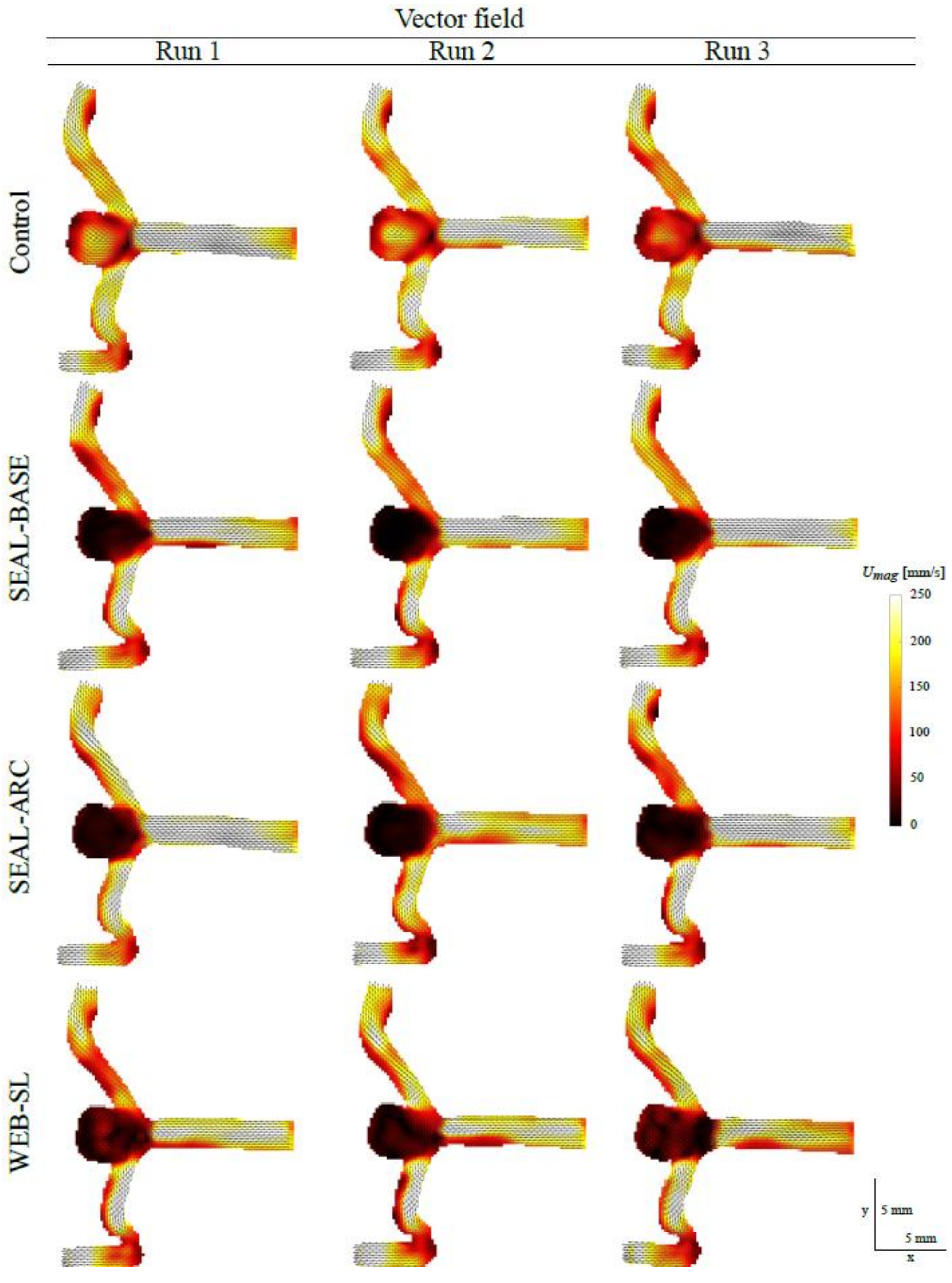

Figure S2: Flow field representation: velocity vectors superimposed over velocity contour colored by the local velocity magnitude for control and deployed devices. The three columns represent the three repetitions of the experiment for each case. Only every other velocity vector is shown in the *x* and *y* directions for better visibility.

Each row corresponds to a different experimental condition, while the three columns display the results from three independent repetitions. For clarity of visualization, every other velocity vector in both the x- and y-directions is plotted.
